# Medical Men’s Per-Centage on Prescriptions

**Published:** 1850-02

**Authors:** 


MEDICAL MEN'S PER CENTAGE ON PRESCRIPTIONS.

Our keen and vigorous contemporary, of the New York Scalpel, makes use of some severe and denunciatory remarks on the practice of those physicians, who accept from apothecaries a per centage on prescriptions, and we fully concur with Dr. Dixon in his condemnation of this disgraceful and degrading practice. We know of nothing that should more promptly expel a man from the intercourse of the medical profession, than such a practice as this. Dr. Dixon says: "In some instances the per centage amounts to one-half! in none, we believe, is it less than a third! certain cabalistic signs being appended to the written prescription to show whether the patient will bear a high charge. What the result of this truly demoniacal arrangement must be, we suppose its bare mention will make apparent to the meanest intellect. For fear, however, any one should not understand us, we simply remark, that the more physic they take, the better for the doctor."

Against this iniquitous practice, we think every man who feels any interest in the elevation of the medical profession, should declare relentless war. It should be put down as all other serious wickedness is, and any man guilty of it should be no more countenanced, than if he were guilty of other heinous offences. It is humiliating to confess the fact, but we have known of instances where it was the apothecary who had too much honor and pride of character to enter into the arrangement we are denouncing. The physician was ready, willing, and urgent. We wish Dr. Dtxon success, in putting down the practice he describes under the title of "Per-centage on Prescriptions." A physician should recommend his patients to send their prescriptions where *they* will be best served, not where *he* will make an addition to his fees.

